# Machine Learning-Based Algorithms for Enhanced Prediction of Local Recurrence and Metastasis in Low Rectal Adenocarcinoma Using Imaging, Surgical, and Pathological Data

**DOI:** 10.3390/diagnostics14060625

**Published:** 2024-03-15

**Authors:** Cristian-Constantin Volovat, Dragos-Viorel Scripcariu, Diana Boboc, Simona-Ruxandra Volovat, Ingrid-Andrada Vasilache, Corina Ursulescu-Lupascu, Liliana Gheorghe, Luiza-Maria Baean, Constantin Volovat, Viorel Scripcariu

**Affiliations:** 1 Department of Radiology, “Grigore T. Popa” University of Medicine and Pharmacy, 700115 Iasi, Romanialiliana.gheorghe@umfiasi.ro (L.G.);; 2 Department of Surgery, “Grigore T. Popa” University of Medicine and Pharmacy, 700115 Iasi, Romania; 3 Department of Medical Oncology-Radiotherapy, “Grigore T. Popa” University of Medicine and Pharmacy, 700115 Iasi, Romania; boboc.diana-ioana@d.umfiasi.ro (D.B.);; 4 Department of Mother and Child Care, “Grigore T. Popa” University of Medicine and Pharmacy, 700115 Iasi, Romania

**Keywords:** rectal cancer, machine learning, risk factors, metastasis, recurrence

## Abstract

(1) Background: Numerous variables could influence the risk of rectal cancer recurrence or metastasis, and machine learning (ML)-based algorithms can help us refine the risk stratification process of these patients and choose the best therapeutic approach. The aim of this study was to assess the predictive performance of 4 ML-based models for the prediction of local recurrence or distant metastasis in patients with locally advanced low rectal adenocarcinomas who underwent neoadjuvant chemoradiotherapy and surgical treatment; (2) Methods: Patients who were admitted at the first Oncologic Surgical Clinic from the Regional Institute of Oncology, Iasi, Romania were retrospectively included in this study between November 2019 and July 2023. Decision tree (DT), naïve Bayes (NB), support vector machine (SVM), and random forest (RF) were used to analyze imagistic, surgical, and pathological data retrieved from the medical files, and their predictive performance was assessed; (3) Results: The best predictive performance was achieved by RF when used to predict disease recurrence (accuracy: 90.85%) or distant metastasis (accuracy: 89.63%). RF was closely followed by SVM (accuracy for recurrence 87.8%; accuracy for metastasis: 87.2%) in terms of predictive performance. NB and DT achieved moderate predictive power for the evaluated outcomes; (4) Conclusions: Complex algorithms such as RF and SVM could be useful for improving the prediction of adverse oncological outcomes in patients with low rectal adenocarcinoma.

## 1. Introduction

Colorectal cancer (CRC) ranks third in terms of frequency of diagnosis and mortality among both men and women in the United States [1]. Nevertheless, it holds the second position in terms of overall cancer-related fatalities and is the primary factor for mortality in males under the age of 50 [1]. Over 50% of all cases and deaths can be attributed to modifiable risk factors, including smoking, a poor diet, excessive alcohol use, a lack of physical activity, and obesity [2].

The 5-year relative survival rate for CRC experienced an increase from 50% in the mid-1970s to 65% throughout the period of 2012–2018 [3,4]. The long-term benefits are a result of the early detection of CRC through routine clinical examinations, and today more precise staging is achieved through advancements in imaging techniques [5,6]. Furthermore, improvements in infection control and surgical techniques, as well as advancements in chemotherapy and radiation, have contributed to these long-term gains [7].

Colorectal cancer ranks as the third most commonly reported cancer in males in Romania, following lung cancer and prostate cancer [8]. This type of neoplasia holds the second position in the list of newly diagnosed cancer cases in women, following breast cancer [3]. In Romania, colorectal cancer is the second most common cause of death connected to cancer, resulting in 4302 deaths in 2020 [3].

Accurate appreciation of the risk of local recurrence and distant metastasis is crucial in determining the appropriateness of neoadjuvant or adjuvant therapy. The literature’s data indicated several clinical, imagistic, surgical, and histopathological risk factors for colorectal cancer recurrence and metastasis. One recent meta-analysis conducted by Xu et al. evaluated 34 risk factors for CRC metastasis and 17 for recurrence from 43 observational studies or meta-analyses [9].

Considering the CRC metastasis as an outcome, the authors outlined 12 risk factors that had a significant effect size [9]. These risk factors included lymphovascular invasion, tumor size greater than 1 cm, tumor budding, poor differentiation, muscularis propria invasion, and extramural vascular invasion (mrEMVI) detected on magnetic resonance imaging (MRI). When the authors investigated the impact of several risk factors for CRC local recurrence, they found a significant effect size of perineural invasion and anastomotic leakage [9].

Another meta-analysis investigated the impact of perioperative blood transfusions on colorectal cancer recurrence in patients undergoing curative resection [10]. The results from this study confirmed perioperative blood transfusions to be independent prognostic factors (odds ratio, OR: 1.42, 95% confidence interval, CI: 1.20–1.67) for the evaluated outcome. Moreover, the effect was observed regardless of timing and in a dose-dependent manner.

Gloor et al. conducted a prospective observational study that evaluated the surgical outcomes and recurrence rates in a cohort of patients with low rectal cancer treated by transanal total mesorectal excision [11]. Their results indicated that a positive distal margin and lymph nodes predicted a local recurrence and distant metastasis of CRC.

Moreover, a retrospective study that evaluated the long-term outcomes of patients with rectal adenocarcinoma who underwent curative surgery indicated that a distal margin ≤ 2 cm, extracapsular invasion of lymph node metastasis, tumor stenosis, and parietal invasion were independent risk factors for recurrence [12].

In recent years, artificial intelligence has gained more interest for its applicability in the prediction of disease occurrence, progression, and/or recurrence [13,14,15]. In the field of oncology, and specifically for the prediction of CRC local recurrence or distant metastasis, several machine learning (ML)-based algorithms or artificial neural networks (ANN) have been developed. Many of these models included the above-mentioned clinical, imagistic, surgical, or histopathological predictors.

Jiang and colleagues tested the predictive performance of an MRI deep learning model for the prediction of survival in patients with rectal cancer [16]. The primary variable used in this model was the segmented tumor volumes obtained from prior treatment T2-weighted MRI images. This model achieved an overall good predictive performance for the evaluated outcome and demonstrated good capacity as a risk stratification tool.

Another retrospective study tested the predictive performance of 4 ML-based models for the prediction of recurrence or metastasis of CRC at 1 year, 3 years, and 5 years milestones [17]. These models were represented by recursive feature elimination (RFE), synthetic minority oversampling technique (SMOTE), and support vector machine (SVM) and used clinical-pathological factors, radiomic features, or their combinations, as predictors. Their results indicated that the combined model performed best for the prediction of CRC metastasis or recurrence at 1 year (area under the curve, AUC = 0.887), 3 years (AUC = 0.813), and 5 years (AUC = 0.794) [17].

One recent study by Skrede et al. performed digital image analysis of more than 12 million histopathological tiles using 10 convolutional neural networks and determined a prognostic biomarker with a hazard ratio (HR) of 3.04 (95%CI: 2.07–4.47; *p* < 0.0001) for the poor prognosis of patients with colorectal cancer [18]. Moreover, Tsai et al. investigated the feasibility of 5 convolutional neural networks for histopathological image classification in patients with colorectal cancers and demonstrated good overall performance [19].

The identification of specific models that achieve the best balance between sensitivity and specificity is a constant challenge, and many efforts should be invested in testing and validation of valuable predictors and their combinations. The aim of this retrospective study was to assess the predictive performance of four ML-based models for the prediction of local recurrence or distant metastasis in patients with locally advanced low rectal adenocarcinomas who underwent neoadjuvant chemoradiotherapy (CRT) and surgical treatment.

## 2. Materials and Methods

This retrospective observational study was conducted at the first Oncologic Surgical Clinic from the Regional Institute of Oncology, Iasi, Romania, between November 2019 and July 2023. Ethical approval for this study was obtained from the Institutional Ethics Committees of the University of Medicine and Pharmacy ‘Grigore T. Popa’ (No. 23103/23 October 2019) and of the Regional Institute of Oncology (No. 245/3 July 2019).

The inclusion criteria comprised patients diagnosed with low rectal tumors (less than 8 cm from the anal verge), a histopathological examination that indicated adenocarcinoma type, who had a preoperative pelvic MRI for staging, who received neoadjuvant therapy and surgical treatment, as well as those who offered their informed consent for participating in this study.

The exclusion criteria comprised patients with rectal cancer who needed emergency surgery, other subtypes of rectal cancer, loss of follow-up, incomplete medical data, or a lack of informed consent.

The following data was recorded: demographic and clinical characteristics, preoperative MRI parameters, histopathological examination of pre-operative biopsies and post-operative specimens, type of surgery, need for blood transfusions, status of total mesolectal excision (TME), postsurgical evolution (local recurrence or distant metastasis), and survival. All patients underwent pelvic MRI examinations on the SIEMENS MAGNETOM Avanto I-class 1.5 Tesla machine (Siemens Healthcare GmbH, Erlangen, Germany).

The examination protocol included the visualization of the pelvis in all three planes:

- Sagittal—this plane is used to locate the tumor and to plan the axial and coronal sequences;
- Axial—the plane is angled perpendicular to the tumor to correctly visualize the extension of the tumor against the rectal wall, as well as the distance between the tumor and the mesorectal fascia (MRF);
- Coronal—the plane is angled parallel to the axis of the tumor, which is perpendicular to the axial series.

The sequences used are the following: multiplanar T2w and T1w which provided valuable morphological information due to the high resolution of anatomical structures. The T2w sequence was ≤ 3 mm thick. The preoperative parameters included: tumor size, location, distance from the anal verge, extramural venous invasion (EMVI) status (tumor invasion into veins beyond muscularis propria), and MRI CRM (mCRM) positivity (tumor within 1 mm of the mesorectal fascia on the scan) [20,21].

Following the elective surgical procedure, the Regional Oncologic Institute’s oncology-trained pathologists evaluated the intestinal specimens in accordance with standard practice. A positive pathological circumferential resection margin (pCRM) was considered to be a distance of less than 1 mm from the tumor cells to the cut specimen margin. Lymph node positivity was also recorded in this stage, and more than 5 positive lymph nodes were considered the cut-off for risk stratification.

Patients were segregated based on the primary outcomes: local recurrence (group 1, *n* = 14 patients) and distal metastasis (group 2, *n* = 24 patients). Patients who did not have local recurrence or distant metastasis were included in the control group (group 3, *n* = 126 patients).

In the first phase of our analysis, we used descriptive statistics and comparison of categorical variables (Pearson’s χ^2^ test) or continuous variables (analysis of variance, ANOVA, followed by Bonferroni posthoc test) between groups.

In the second stage of the analysis, we used a Cox regression model with the Breslow method for ties to identify imaging, surgical, and pathological predictors for CRC recurrence or metastasis, and quantified their impact as a hazard ratio (HR) and 95% CI. A *p*-value less than 0.05 was considered statistically significant. These analyses were performed using STATA SE (version 17, 2023, StataCorp LLC, College Station, TX, USA).

In the third stage of the analysis, predictors which had a significant effect on the evaluated outcomes were included in 4 machine-learning-based models: decision tree (DT), naïve Bayes (NB), support vector machine (SVM), and random forest (RF). The database was divided into two sets: 70% for testing and 30% for training. Most machine-learning-based studies choose this configuration, especially for small datasets such as ours [13,14,15,22].

Additionally, a 5-fold cross-validation was performed. The predictive performance of these models was tested for the evaluated outcomes using a sensitivity analysis. The following parameters were reported: sensibility (Se), specificity (Sp), false positive rate (FPR), accuracy, AUC value, Matthews correlation coefficient, and F1 score.

Sensitivity is defined as the number of true positives relative to the total number of sick individuals in the population. It is the probability of a positive test given that the patient is truly positive [23]. Specificity is expressed as the number of true negatives relative to the total number of healthy individuals [23].

FPR was defined as 1- specificity, while accuracy was defined as the sum of true positives and true negatives relative to the total population [24,25]. Mathews coefficient is a correlation coefficient between the observed and predicted binary classifications, that ranges between −1 and +1 [26].

The F1 score was considered the harmonic mean of precision and recall [27]. The models were constructed and analyzed using Matlab (version R2023a, The MathWorks, Inc., Natick, MA, USA).

## 3. Results

A total of 164 patients with low rectal adenocarcinoma who underwent neoadjuvant therapy and surgical treatment were included in the study, and their clinical and paraclinical characteristics are presented in Table 1.

All examined groups were similar regarding their age (*p* = 0.88), gender distribution (*p* = 0.92), medium of living (*p* = 0.48), BMI (*p* = 0.96), and smoking habit (*p* = 0.86), and no statistically significant difference between groups was found considering these characteristics.

On the other hand, the univariate analysis indicated that all imagistic parameters were significantly different between groups (*p* < 0.001). Thus, patients who later developed local recurrence or CRC metastasis presented with significantly higher rates of mCRM and EMVI positivity, as well as a higher incidence of anteriorly located tumors at a distance of less than 4 cm from the anal verge.

Moreover, the proportion of Hartmann procedures and pelvic exenterations was significantly higher for patients from the first and second groups compared with controls (*p* = 0.04). The same groups experienced significantly higher rates of incomplete TME (*p*= 0.005) and blood transfusions (*p* < 0.001) compared with controls.

Regarding histopathological characteristics, both a high number of positive lymph nodes and positive pCRM were significantly more frequently encountered in the groups of patients who later developed local recurrence or CRC metastasis (*p* < 0.001).

Table 2 comprises the results from the Cox regression model, which used the local recurrence of CRC as the outcome, and significant parameters in the univariate analysis as predictors.

A positive pCRM (HR: 53.33, 95%CI: 11.37–249.97, *p* < 0.001), mrEMVI (HR: 28.56, 95%CI: 6.15–132.62, *p* < 0.001), and more than 5 positive lymph nodes (HR: 13.68, 95%CI: 3.98–46.97, *p* < 0.001) demonstrated the highest positive impact on the occurrence of CRC local recurrence.

Also, mCRM (*p* = 0.002), the anterior location of the tumor (*p* = 0.005), and an incomplete TME (*p* = 0.022) had a significant impact on the evaluated outcome’s occurrence, even though it was more reduced compared to the previous predictors.

On the other hand, a distance of less than 4 cm from the anal verge (*p* = 0.230) and the need for blood transfusions (*p* = 0.078) did not appear to have a significant positive impact on the evaluated outcome.

Table 3 comprises the results from a Cox regression model that used the distant metastasis of CRC as the outcome and significant parameters in the univariate analysis as predictors.

Our model showed that more than 5 positive lymph nodes (HR: 48.02, 95%CI: 16.74–137.70, *p* < 0.001), mrEMVI (HR: 23.76, 95%CI: 8.98–62.88, *p* < 0.001), mCRM (HR: 21.22, 95%CI: 7.06–63.77, *p* < 0.001), the anterior location of the tumor (HR: 13.40, 95%CI: 5.12–35.07, *p* < 0.001), a distance of less than 4 cm from the anal verge (HR: 7.43, 95%CI: 2.48–22.24, *p* < 0.001), and a positive pCRM (HR: 15.41, 95%CI: 6.26–37.93, *p* < 0.001) significantly increased the risk of CRC distant metastasis.

Also, incomplete TME (*p* = 0.002) and the need for blood transfusions (*p* = 0.026) had a smaller, but significant, impact on the risk of CRC metastasis.

**Figure 1:**
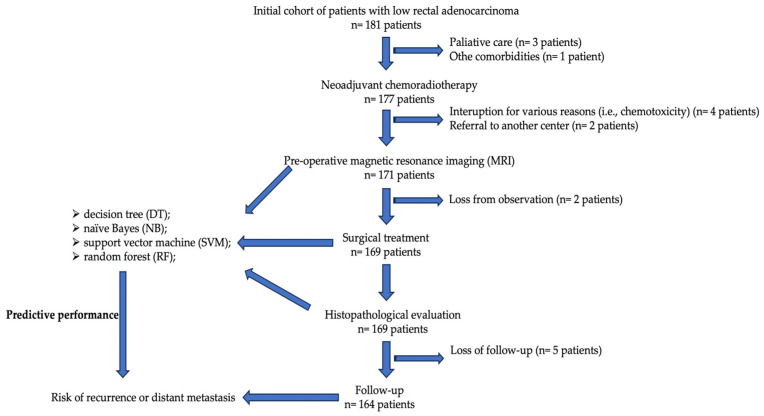
Flowchart with the study methodology.

**Figure 2:**
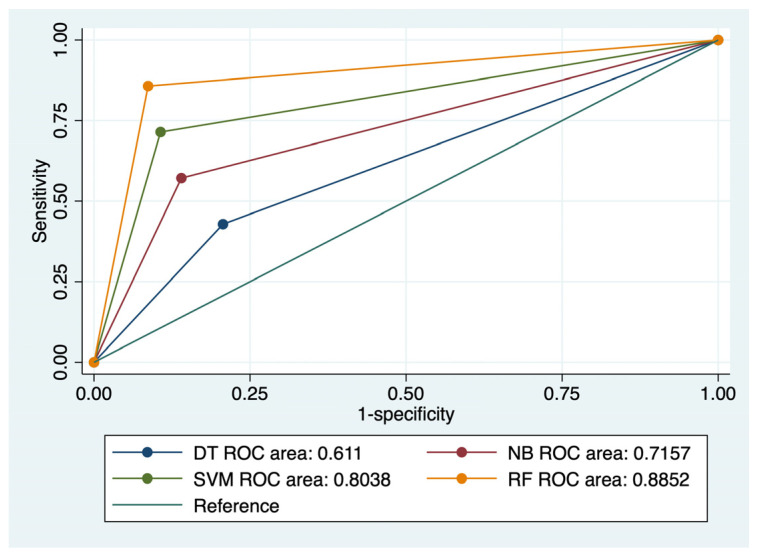
Comparison of ROC curves corresponding to 4 models used for the prediction of CRC local recurrence.

**Figure 3:**
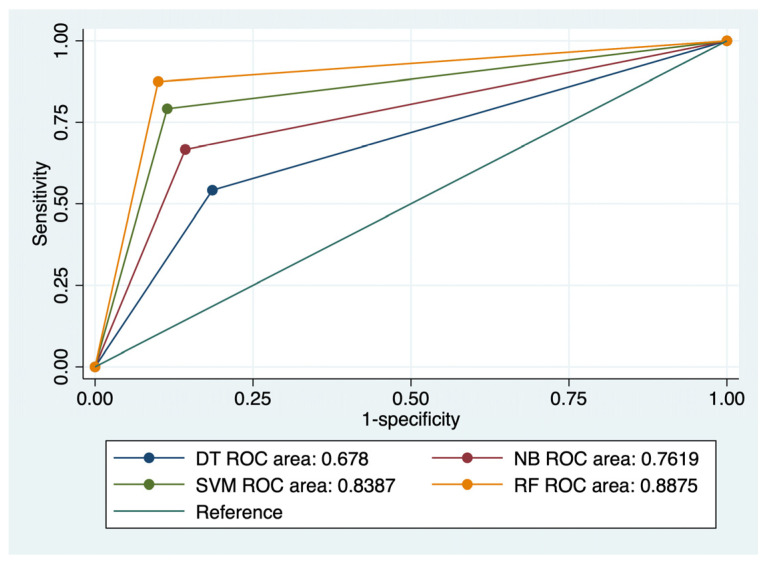
Comparison of ROC curves corresponding to 4 models used for the prediction of CRC distant metastasis.

Significant predictors from the Cox hazard regression were included in 4 machine-learning-based algorithms, and their predictive performance for CRC local recurrence and distant metastasis was calculated (Table 4). A flowchart with the study methodology is presented in Figure 1. Comparisons of the ROC curves corresponding to the evaluated models and outcomes are presented in Figure 2 and Figure 3.

RF achieved the best results in terms of prediction of CRC local recurrence: Se- 85.71%, Sp- 91.33%, FPR- 8%, and accuracy of 90.85%. This type of algorithm also best predicted CRC distant metastasis, with a Se of 87.5%, Sp of 90%, FPR of 1%, and an accuracy of 89.63%. RF was closely followed by SVM (accuracy for recurrence 87.8%; accuracy for metastasis: 87.2%) in terms of predictive performance. NB and DT achieved moderate predictive power for the evaluated outcomes.

The ROC comparisons outlined RF and SVM models as having the highest AUC value for the prediction of CRC recurrence (AUC: 0.885/AUC: 0.803) and distal metastasis (AUC: 0.887/AUC: 0.838) (Figure 2 and Figure 3). NB performed better than DT for prediction of the evaluated outcomes: AUC values for recurrence—0.715 versus 0.611, and AUC values for metastasis—0.761 versus 0.678.

## 4. Discussion

Regular screening and prompt diagnostics of colorectal cancer have reduced the incidence of tumors in the early stages. Moreover, it was observed that in 2019, 60% of newly reported cases were classified as advanced, compared to 52% in the mid-2000s and 57% in 1995, prior to the implementation of universal screening [28]. Additionally, there has been a trend toward an increase in left-sided tumors, including rectal cancer, which accounted for 27% of cases in 1995 and rose to 31% in 2019 [28]. This epidemiological context outlines the need to identify the best prognostic factors that are associated with a high risk of local recurrence or distant metastasis from locally advanced rectal cancers.

This study was focused on patients diagnosed with low rectal adenocarcinomas, which is the most frequent type of rectal cancer (accounting for approximately 96% of all CRCs.) [29]. Specifically, we identified risk factors that increase the odds of rectal cancer local recurrence or distant metastasis in this cohort of patients, and we included the significant predictors in four machine-learning models whose predictive performance was determined.

Our results indicated that both local recurrence and distant metastasis shared common significant predictors such as positivity for pCRM, mCRM, and mrEMVI, as well as more than 5 positive lymph nodes, an anterior location of the tumor, and an incomplete TME. Additionally, the following predictors increased the risk of distant metastasis: a distance of less than 4 cm from the anal verge, and the need for blood transfusions.

These predictors were also identified in the literature to increase the risk of adverse outcomes recurrence. For example, Ma et al. found in a prospective study on a cohort of 209 patients diagnosed with rectal cancer who underwent tumor resection that mCRM was an independent risk factor for local recurrence, with an HR of 3.49 (*p* = 0.003) [30]. Currently, there is no evidence supporting the utilization of re-radiation for a positive pCRM after undergoing preoperative radiotherapy and TME surgery. The presence of a positive circumferential resection margin following preoperative radiotherapy and surgery creates ambiguity regarding the function of post-operative radiotherapy [31]. Therefore, our center does not commonly practice it due to the heightened toxicity and uncertain survival benefits.

Another study investigated the influence of a pCRM in 3196 individuals diagnosed with rectal cancer who underwent complete TME, with or without radiotherapy [32]. Their results indicated that a positive mCRM was associated with an increased risk of tumor recurrence (HR: 4.18, 95%CI: 2.48–7.05) and distant metastasis (HR: 2.81, 95%CI: 1.93–4.09) in patients who underwent only TME. Moreover, the same parameter was also associated with an increased risk of local recurrence (HR: 0.53, 95%CI: 0.30–0.90) and distal metastasis (HR: 1.22, 95%CI: 0.86–1.61) in patients who underwent TME and radiotherapy [32].

A recent meta-analysis was conducted to assess the prognostic significance of magnetic resonance extramural vascular invasion (mrEMVI) in predicting unfavorable oncologic outcomes in patients who had neoadjuvant therapy followed by total mesorectal excision [33]. The results of this meta-analysis confirmed mrEMVI as an independent prognostic factor for recurrence, metastasis, and decreased disease-free survival. Moreover, the GEMCAD 0801 trial investigated the prognostic value of mrENVI and other predictors for adverse oncological outcomes in patients who underwent primary chemotherapy for rectal cancer [34]. A positive mrEMVI was associated with an increased risk of local recurrence in a 3-year time frame (HR: 9.220, 95%CI: 0.802–105.965).

Lymph node positivity was considered an important independent risk factor for CRC recurrence or metastasis, but the cut-off differs between studies. Peng et al. conducted a retrospective analysis on the prognostic value of LNR (ratio of metastatic to retrieved lymph nodes) in patients with node-positive rectal cancer, who were treated with curative anterior resection, over a 14-year period [35]. Their results indicated that the five-year local recurrence rate was significantly higher in patients with an LNR between 0.14 and 1 (3.6% in LNR < 0.14 versus 15.6% in LNR 0.14–1, *p* = 0.019). Another retrospective study aimed to identify the prognostic significance of lateral node metastases in patients with stage 3 or pT4 low rectal adenocarcinoma [36]. The results from this study indicated that patients with lateral node metastases had a significantly shorter postoperative survival (5-year survival rate of 42 versus 71.6%; *p* < 0·001) and an increased risk of local recurrence (44 versus 11.7%; *p* < 0·001) in comparison with controls.

Finally, the anterior location of the tumor [37,38], an incomplete TME [39,40], a distance of less than 4 cm from the anal verge [41,42], and the need for blood transfusions [43,44] were also cited in the literature as independent prognostic factors for the local recurrence and/or distant metastasis of rectal cancer. Thus, in the second stage of the analysis, all these significant predictors were included in the following ML-based models: DT, NB, SVM, and RF, whose predictive performance was calculated.

Our results indicated that the best predictive performance was achieved by RF when used to predict low rectal adenocarcinoma recurrence or distant metastasis, with corresponding accuracies of 90.85% and 89.63%. RF was closely followed by SVM (accuracy for recurrence: 87.8%; accuracy for metastasis: 87.2%) in terms of predictive performance. NB and DT achieved moderate predictive power for the evaluated outcomes. These results are in line with previously published literature, although data is scarce and variable when considering specific machine learning-based algorithms.

Jeon et al. investigated the predictive performance of 4 ML-based algorithms (logistic regression, LR, SVM, RF, and extreme gradient boosting, XGBoost) based on clinical and paraclinical data for the prediction of rectal cancer recurrence after curative resection [45]. Their results indicated that SVM achieved the best predictive performance (AUC: 0.831, Se: 69.2%, Sp: 81.4%, and accuracy: 79.8%) for the prediction of the evaluated outcome. RF, which achieved a Se of 73.1%, a Sp of 80.2%, an accuracy of 79.3%, and an AUC value of 0.826, closely followed this algorithm [45]. In contrast, the lowest AUC value was obtained for the XGBoost method (0.804), with a sensitivity, specificity, and accuracy of 30.8%, 92.8%, and 84.5%, respectively.

Xu et al. investigated the predictive performance of 4 ML-based algorithms (LR, DT, GradientBoosting, and lightGBM) for the prediction of the postoperative recurrence risk in patients diagnosed with stage IV colorectal cancer [46]. Their results indicated that the GradientBoosting model (AUC: 0.734) and the lightGBM model (F1_score: 0.974) performed better than LR (AUC: 0.692) and DT in the testing phase. On the other hand, a study that included only radiomic features in SVM or LR models indicated that LR models achieve superior predictive performance (accuracy: 80%, Se: 83%, Sp: 76%, and AUC: 0.87) [47].

### Limitations and Future Work Recommendations

The results from this study should be interpreted considering the following limitations: the small cohort of patients, the limited time frame for patients’ follow-up, and the small number of parameters evaluated. On the other hand, this study has the advantage of testing various ML algorithms for the prediction of adverse oncological outcomes in low rectal cancer patients.

We hypothesize that further studies, on a larger cohort of patients, could include several panels of clinical and paraclinical parameters in several machine learning algorithms or convolutional neural networks in order to better establish their predictive performance. These approaches allow better image segmentation or feature discrimination, and allow the analysis of a large dataset, even with high rates of missing data [48,49].

Moreover, some studies have outlined the potential of deep learning techniques to include a wide range of parametric and non-parametric data and to create a bridge between multiple areas of research, with practical implications in the treatment planning of oncologic patients [50,51].

This paper outlined several clinical and paraclinical risk factors that were associated with an increased risk of rectal adenocarcinoma recurrence or metastasis. These risk factors could be further included in various risk stratification algorithms, either classical or based on machine learning.

Moreover, we tested four supervised machine learning algorithms, which included these predictors, for their predictive power. Their performance indicated the need for further refinement and/or inclusion of additional data, as their accuracies ranged between 76.22 and 90.85%.

This study tested the predictive performance of machine learning algorithms on a small cohort of patients. This study cohort had the particularity of including locally advanced rectal adenocarcinomas who underwent neoadjuvant therapy and surgical treatment, and this could be an explanation for the small number of cases included. The number of cases included in studies that evaluated the predictive performance of machine learning algorithms for rectal cancer recurrence or metastasis is variable, depending on numerous factors.

For example, Sluckin et al. investigated the predictive performance of a deep learning model based on imaging and clinical features in 196 patients diagnosed with rectal cancer from three tertiary centers in the Netherlands [52]. The authors showed that their model achieved an AUC value of 0.78 for the prediction of lateral recurrence and 0.80 for the prediction of ipsi-lateral local recurrences.

Another study evaluated the performance of deep learning radiomics for the prediction of distant metastasis in 235 patients with locally advanced rectal cancer from three hospitals and demonstrated that their model achieved an AUC value of 0.894 in the validation phase [53].

Moreover, Liang et al. tested the predictive performance of two machine learning algorithms, SVM and logistic regression, for the prediction of metachronous liver metastases in a cohort of 108 patients diagnosed with rectal cancer, and demonstrated the superior performance of the logistic regression algorithm, which achieved an AUC value of 0.87 for the evaluated outcome [47].

Machine learning-based algorithms and neural networks are increasingly used in the medical field, and we hypothesize that their inclusion in clinical practice could serve as useful instruments for risk stratification and prognosis in various clinical scenarios.

This study outlines the efficacy of machine learning algorithms that operate with clinical and paraclinical data for the prediction of rectal adenocarcinoma local recurrence and distant metastasis in a cohort of patients from Romania. We hypothesize that further prospective internal and external validation of our models could aid the update of our oncological and surgical perspective regarding the profile of high-risk patients for adverse oncological outcomes and prognosis.

## 5. Conclusions

Machine learning is a complex scientific field that has gained a lot of interest in personalized medicine. Finding the best formula for disease recurrence or metastasis prediction is a constant challenge, and numerous predictors should be carefully evaluated for their prognostic value before including them in specific formulas.

This retrospective study indicated that several clinical and paraclinical predictors have a significant impact on rectal cancer local recurrence or metastasis, and their inclusion in 4 ML-based algorithms indicated overall good predictive performance.

The highest predictive performance for the evaluated outcomes was achieved by RF, which was closely followed by SVM. NB and DT achieved moderate predictive power for the evaluated outcomes.

The inclusion of machine learning algorithms in clinical practice could serve as useful instruments for risk stratification and prognosis in various clinical scenarios.

## Figures and Tables

**Table 1:** Demographic and clinical characteristics of the evaluated groups.

Variable	Local Recurrence (Group 1, *n* = 14 Patients)	Distant Metastasis (Group 2, *n* = 24 Patients)	Control Group (Group 3, *n* = 126 Patients)	*p* Value
Age, years (mean ± SD)	68.57 ± 8.77	65.87 ± 9.24	63.06 ± 9.26	0.88
Gender (*n*/%)	Male = 9 (64.28%) Female = 5 (35.71%)	Male = 14 (58.3%) Female = 10 (41.66%)	Male = 78 (61.90%) Female = 48 (38.09%)	0.92
Medium (*n*/%)	Urban = 8 (57.14%) Rural = 6 (42.85%)	Urban = 10 (41.66%) Rural = 14 (58.33%)	Urban = 51 (40.47%) Rural = 75 (59.52%)	0.48
BMI, kg/m^2^ (mean ± SD)	25.76 ± 4.31	25.28 ± 3.69	24.82 ± 4.12	0.96
Smoking (*n*/%)	Yes = 3 (21.42%)	Yes = 4 (16.66%)	Yes = 20 (15.87%)	0.86
Positive mCRM (*n*/%)	Yes = 9 (64.29%)	Yes = 19 (79.17%)	Yes = 21 (16.66%)	<0.001
Positive mrEMVI (*n*/%)	Yes = 12 (85.71%)	Yes = 20 (83.33%)	Yes = 12 (9.52%)	<0.001
Anterior location (*n*/%)	Yes = 8 (57.14%)	Yes = 17 (70.83%)	Yes = 11 (8.73%)	<0.001
Distance from anal verge (*n*/%)	<4 cm = 7 (50%) >4 cm = 7 (50%)	<4 cm = 19 (79.17%) >4 cm = 5 (20.83%)	<4 cm = 35 (27.77%) >4 cm = 91 (72.22%)	<0.001
Type of surgery (*n*/%)	AP resection = 8 (57.14%) Hartmann procedure = 3 (21.42%) Anterior resection = 1 (7.14%) Pelvic exenteration = 2 (14.28%)	AP resection = 15 (62.5%) Hartmann procedure = 6 (25%) Anterior resection = 1 (4.16%) Pelvic exenteration = 2 (7.69%)	AP resection = 98 (77.77%) Hartmann procedure = 19 (15.07%) Anterior resection = 8 (6.34%) Pelvic exenteration = 1 (0.79%)	0.04
Incomplete TME (*n*/%)	Yes = 6 (42.85%)	Yes = 9 (37.5%)	Yes = 15 (11.90%)	0.0005
Need for blood transfusions (*n*/%)	Yes = 5 (35.71%)	Yes = 13 (54.16%)	Yes = 16 (12.69%)	<0.001
More than 5 positive lymph nodes (*n*/%)	Yes = 9 (64.29%)	Yes = 18 (75%)	Yes = 13 (10.31%)	<0.001
Positive pCRM (*n*/%)	Yes = 11 (78.57%)	Yes = 14 (58.33%)	Yes = 6 (4.76%)	<0.001

Table legend: pCRM—pathological (positive) circumferential resection margin; mCRM—positive circumferential resection margin on MRI; mrEMVI—extramural venous invasion on MRI; SD—standard deviation; BMI—body mass index; AP—abdominoperineal resection; TME—total mesorectal excision.

**Table 2:** Results from the Cox regression model for evaluating the impact of imagistic, surgical, and histopathological predictors on the CRC local recurrence.

Predictors	HR	Standard Error	95% CI (Lower Bound and Upper Bound)	*p* Value
mCRM	7.26	4.55	2.12–24.85	0.002
mrEMVI	28.56	22.37	6.15–132.62	<0.001
Anterior location	5.55	3.36	1.69–18.23	0.005
Less than 4 cm from anal verge	2.06	1.25	0.63–6.77	0.230
Incomplete TME	4.21	2.64	1.23–14.42	0.022
Need for blood transfusions	3.29	2.23	0.87–12.43	0.078
More than 5 positive lymph nodes	13.68	8.61	3.98–46.97	<0.001
Positive pCRM	53.33	42.03	11.37–249.97	<0.001

Table legend: HR—hazard ratio; CI—confidence interval; mCRM—positive circumferential resection margin on MRI; mrEMVI—extramural venous invasion on MRI; pCRM—pathological circumferential resection margin; TME—total mesorectal excision.

**Table 3:** Results from the Cox regression model for evaluating the impact of imagistic, surgical, and histopathological predictors on CRC distal metastasis.

Predictors	HR	Standard Error	95% CI (Lower Bound and Upper Bound)	*p* Value
mCRM	21.22	11.91	7.06–63.77	<0.001
mrEMVI	23.76	11.79	8.98–62.88	<0.001
Anterior location	13.40	6.57	5.12–35.07	<0.001
Less than 4 cm from anal verge	7.43	4.15	2.48–22.24	<0.001
Incomplete TME	4.32	2.03	1.71–10.86	0.002
Need for blood transfusions	3.16	1.63	1.14–8.72	0.026
More than 5 positive lymph nodes	48.02	25.81	16.74–137.70	<0.001
Positive pCRM	15.41	7.08	6.26–37.93	<0.001

Table legend: HR—hazard ratio; CI—confidence interval; mCRM—positive circumferential resection margin on MRI; mrEMVI—extramural venous invasion on MRI; pCRM—pathological circumferential resection margin; TME—total mesorectal excision.

**Table 4:** Predictive performance of ML-based algorithms for the prediction of CRC local recurrence and distal metastasis.

ML Model	Groups	Se (%)	SP (%)	FPR (%)	Matthews Coefficient	Accuracy (%)	AUC Value	F1 Score
DT	Recurrence (14 patients)	42.86	79.33	20.66	0.14	76.22	0.611	0.23
	Metastasis (24 patients)	54.17	81.43	18.57	0.29	77.4	0.678	0.41
NB	Recurrence (14 patients)	57.14	86	14	0.31	83.54	0.715	0.37
	Metastasis (24 patients)	66.67	85.71	14.2	0.44	82.93	0.761	0.53
SVM	Recurrence (14 patients)	71.43	89.33	10.6	0.46	87.8	0.803	0.5
	Metastasis (24 patients)	79.17	88.57	11.42	0.58	87.2	0.838	0.64
RF	Recurrence (14 patients)	85.71	91.33	8	0.59	90.85	0.885	0.61
	Metastasis (24 patients)	87.5	90	1	0.66	89.63	0.887	0.71

Table legend: DT—decision trees, NB—naïve Bayes, SVM—support vector machine, RF—random forest, Se—sensibility; SP—specificity, FPR—False positive rate; AUC—area under the curve.

## Data Availability

The datasets are available from the correspondent authors upon a reasonable request due to local policies.
